# H_2_O_2_‐induced microvessel barrier dysfunction: the interplay between reactive oxygen species, nitric oxide, and peroxynitrite

**DOI:** 10.14814/phy2.14206

**Published:** 2019-08-25

**Authors:** Xueping Zhou, Yan Qian, Dong Yuan, Qilong Feng, Pingnian He

**Affiliations:** ^1^ Department of Physiology and Pharmacology, School of Medicine West Virginia University Morgantown West Virginia; ^2^ Department of Cellular and Molecular Physiology, College of Medicine Penn State University Hershey Pennsylvania

**Keywords:** Endothelial [Ca^2+^]_i_, H_2_O_2_, nitric oxide, microvessel permeability, peroxynitrite

## Abstract

Elevated H_2_O_2_ is implicated in many cardiovascular diseases. We previously demonstrated that H_2_O_2_‐induced endothelial nitric oxide synthase (eNOS) activation and excessive NO production contribute to vascular cell injury and increases in microvessel permeability. However, the mechanisms of excessive NO‐mediated vascular injury and hyperpermeability remain unknown. This study aims to examine the functional role of NO‐derived peroxynitrite (ONOO^−^) in H_2_O_2_‐induced vascular barrier dysfunction by elucidating the interrelationships between H_2_O_2_‐induced NO, superoxide, ONOO^−^, and changes in endothelial [Ca^2+^]_i_ and microvessel permeability. Experiments were conducted on intact rat mesenteric venules. Microvessel permeability was determined by measuring hydraulic conductivity (Lp). Endothelial [Ca^2+^]_i_, NO, and O_2_^−^ were assessed with fluorescence imaging. Perfusion of vessels with H_2_O_2_ (10 µmol/L) induced marked productions of NO and O_2_^−^, resulting in extensive protein tyrosine nitration, a biomarker of ONOO^−^. The formation of ONOO^−^ was abolished by inhibition of NOS with N^G^‐Methyl‐L‐arginine. Blocking NO production or scavenging ONOO^−^ by uric acid prevented H_2_O_2_‐induced increases in endothelial [Ca^2+^]_i_ and Lp. Additionally, the application of exogenous ONOO^−^ to microvessels induced delayed and progressive increases in endothelial [Ca^2+^]_i_ and microvessel Lp, a pattern similar to that observed in H_2_O_2_‐perfused vessels. Importantly, ONOO^−^ caused further activation of eNOS with amplified NO production. We conclude that the augmentation of NO‐derived ONOO^−^ is essential for H_2_O_2_‐induced endothelial Ca^2+^ overload and progressively increased microvessel permeability, which is achieved by self‐promoted amplifications of NO‐dependent signaling cascades. This novel mechanism provides new insight into the reactive oxygen and/or reactive nitrogen species‐mediated vascular dysfunction in cardiovascular diseases.

## Introduction

Increased production of reactive oxygen species (ROS) has been demonstrated to cause impaired vascular barrier function, resulting in tissue damage and organ dysfunction (Del Maestro et al., 1981; Cai and Harrison, 2000; Zhu and He, 2006; Zhou et al., 2009; Di et al., 2016; Mundi et al., 2017). Our previous studies demonstrated that hydrogen peroxide (H_2_O_2_) at a pathophysiologcally relevant concentration (10 μmol/L) induced delayed and progressive increases in endothelial calcium concentration (EC [Ca^2+^]_i_) and microvessel permeability that was attributed to H_2_O_2_‐induced excessive nitric oxide (NO) production through endothelial nitric oxide synthase (eNOS) activation (Zhou et al., 2013).

While physiological levels of NO have been recognized to be essential for the maintenance of many vascular functions, excessive NO has been demonstrated to play important roles in mediating inflammation‐induced vascular leakage (Yuan et al., 1993; He et al., 1997; Zhu and He, 2005; Hatakeyama et al., 2006; Zhou and He, 2010; Zhou and He, 2011; Mundi et al., 2017). Our previous studies demonstrated that, instead of causing immediate and transient increases in microvessel permeability as observed in inflammatory mediator‐stimulated vessels (He et al., 1996; Baluk et al., 1997; Zhu and He, 2005; Jiang et al., 2008; Zhou and He, 2011), large amounts of H_2_O_2_‐induced NO production resulted in caspase activation and impairment of Ca^2+^ homeostasis, leading to vascular cell apoptosis, and delayed and progressive increases in microvessel permeability (Zhou et al., 2013).

Exogenously supplied NO has been reported to have potential cytotoxic effects in cultured endothelial cells (Shen et al., 1998; Shimizu et al., 1998). However, the mechanisms of NO‐mediated vascular cell injury, particularly in H_2_O_2_‐stimulated intact vessels remain unknown. Accumulating evidence suggest that the cytotoxic effects of NO are most likely mediated by its oxidation product, particularly peroxynitrite (ONOO^−^), rather than NO itself (Xia et al., 1996; Riobo et al., 2001; Pacher et al., 2007). NO reacts rapidly with superoxide (O_2_^−^) at a diffusion‐controlled rate (~1 × 10^10^ M^−1^ s^−1^) to form ONOO^−^, a strong oxidant capable of oxidizing and nitrating lipid, protein, and DNA, resulting in cell death and tissue injury (Radi et al., 1991; Pacher et al., 2007; Szabo et al., 2007). Recently, H_2_O_2_ has been shown to induce further production of O_2_^−^ and H_2_O_2_ mainly through activation of NAD(P)H oxidase (Li et al., 2001; Cai, 2005), which is considered as an important mechanism for ROS‐mediated pathogenesis of cardiovascular diseases. However, whether the interplay between reactive oxygen species, nitric oxide, and peroxynitrite is accountable for H_2_O_2_‐induced microvessel barrier dysfunction remains unknown.

Currently, the role of ONOO^−^ in the regulation of eNOS activity and NO production, particularly under in vivo conditions, has not been well defined. While some early studies reported that ONOO^−^ induced vasodilation through increased NO production (Liu et al., 1994; Villa et al., 1994), others on cultured bovine endothelial cells suggested that, although ONOO^−^ increased eNOS activity through enhanced phosphorylation at Ser^1179^, it increased ${O_{2}}^{\cdot -}$ production, instead of NO (Zou et al., 2002; Zou et al., 2002). The interrelationship between ONOO^−^ and eNOS activity and their roles in the regulation of vascular barrier function in intact vessels has not been well explored.

This study is designed to examine the functional roles of ONOO^−^ in H_2_O_2_‐induced microvessel barrier dysfunction by elucidating the interrelationships between H_2_O_2_, eNOS‐derived NO, O_2_^−^, ONOO^−^, and the changes in EC [Ca^2+^]_i_ and microvessel permeability. Experiments were conducted on individually perfused mesenteric venules with intact surrounding circulation. EC [Ca^2+^]_i_ and NO were measured in Fura‐2 and DAF‐2‐loaded vessels, respectively. Microvessel permeability was determined by measuring hydraulic conductivity (Lp). We first measured H_2_O_2_‐induced changes in EC [Ca^2+^]_i_, NO production, and microvessel Lp. Under the same experimental conditions, H_2_O_2_‐induced O_2_^−^ production was assessed using dihydroethidium. The formation of ONOO^−^ was examined by fluorescent immunostaining of nitrotyrosine, a biomarker of ONOO^−^, in H_2_O_2_‐perfused vessels. The functional relationship between ONOO^−^, and changes in EC [Ca^2+^]_i_ and microvessel permeability was further investigated using uric acid, an endogenous ONOO^−^ scavenger. The direct effects of exogenously applied ONOO^−^ on EC [Ca^2+^]_i_, NO production, as well as microvessel Lp, were also examined.

## Materials and Methods

### Animal preparation

All animal experiments were conducted on female Sprague–Dawley rats (2–3 months old, 220–250 g; Hilltop Laboratory Animal, Scottdale, PA). All procedures and animal use were approved by the Animal Care and Use Committee at West Virginia University. Rats were anesthetized with pentobarbital sodium (65 mg/kg body wt) administered subcutaneously. A midline surgical incision (1.5–2 cm) was made in the abdominal wall and the mesentery was gently taken out from the abdominal cavity and spread over a glass coverslip attached to an animal tray for studies. The upper surface of the mesentery was continuously superfused with mammalian Ringer's solution at 37°C. Each experiment was performed on one microvessel per animal.

### Measurement of Lp in individually perfused rat mesenteric microvessels

Microvessel permeability was assessed by measuring hydraulic conductivity, Lp, using modified Landis technique, which measures the volume flux of water across the microvessel wall. Details have been described previously (Curry and Sarelius, 1983; Kendall and Michel, 1995; He et al., 1996). Briefly, a single venular microvessel with diameters ranging between 35 and 50 μm was cannulated with a micropipette and perfused with albumin‐Ringer solution (control) containing 1% (vol/vol) hamster red blood cells as markers. A known hydrostatic pressure (40–60 cmH_2_O), controlled by a water manometer, is applied through the micropipette to the vessel lumen, which allows the perfusate to continuously flow through the vessel. For each measurement, the perfused vessel was occluded briefly downstream with a glass rod. The initial water flux/unit area of microvessel wall (Jv/A) was calculated from the velocity of the marker cell after vessel occlusion, the vessel radius, and the distance between the marker cell and the occlusion site. Lp was calculated as the slope of the relationship between Jv/A and the pressure difference across the vessel wall. In each experiment, the baseline Lp and the Lp after the application of testing solutions were measured in the same vessel, and the changes in Lp were expressed as the ratio of Lp_test_/Lp_control_. All testing agents were added to the perfusate and delivered into the vessel lumen through the cannulation pipette. To prevent the marker red blood cells from interacting with H_2_O_2_ and ONOO^−^ in the perfusate, marker cells were absent during the perfusion period and added back for Lp measurements after designated duration of perfusion.

### Measurements of EC [Ca^2+^]_i_

EC [Ca^2+^]_i_ was measured in individually perfused microvessels using the fluorescent Ca^2+^ indicator fura 2‐AM. Experiments were performed on a Nikon Diaphod 300 microscope equipped with a Nikon photometry system. In each experiment, a venular microvessel was cannulated and perfused first with albumin‐Ringer solution that contained 10 µmol/L of fura 2‐AM for 45 min. The vessel was then recannulated and perfused with albumin‐Ringer solution for 10 min to remove fura 2‐AM from the vessel lumen. A segment of fura 2‐AM‐loaded vessel at least 100 µm away from the cannulation site was then positioned within the measuring window which covered ~50 ECs forming the vessel wall. The excitation wavelengths for fura 2‐AM were selected by two narrow‐band interference filters (340 ± 5 and 380 ± 5 nm; Oriel), and the emission was separated with a dichroic mirror (DM400) and a wide‐band interference filter (500 ± 35 nm; Oriel). The corresponding FI values (FI_340_ and FI_380_, respectively) were collected with a 0.25‐sec exposure at each wavelength. Baseline EC [Ca^2+^]_i_ was measured after fura 2‐AM was removed from the vessel lumen with albumin‐Ringer perfusion for 10 min. Then the same vessel was recannulated with pipette containing test reagents (e.g., H_2_O_2,_ ONOO^−^, L‐NMMA, uric acid, LaCl_3,_ etc.) and the changes in EC [Ca^2+^]_i_ were measured immediately after the recannulation. At the end of the experiment, the microvessel was superfused with a modified Ringer solution (5 mmol/L of Mn^2+^ without Ca^2+^) and perfused with the same solution that contained ionomycin (10 µmol/L) to bleach the Ca^2+^‐sensitive form of fura 2. The background FI due to unconverted fura 2‐AM and other Ca^2+^‐insensitive forms of fura 2 were subtracted from FI_340_ and FI_380_ values. The ratios of the two FI values were converted to Ca^2+^ concentrations using an in vitro calibration curve (He et al., 1996).

### Measurement of endothelial NO production

**Figure 1:**
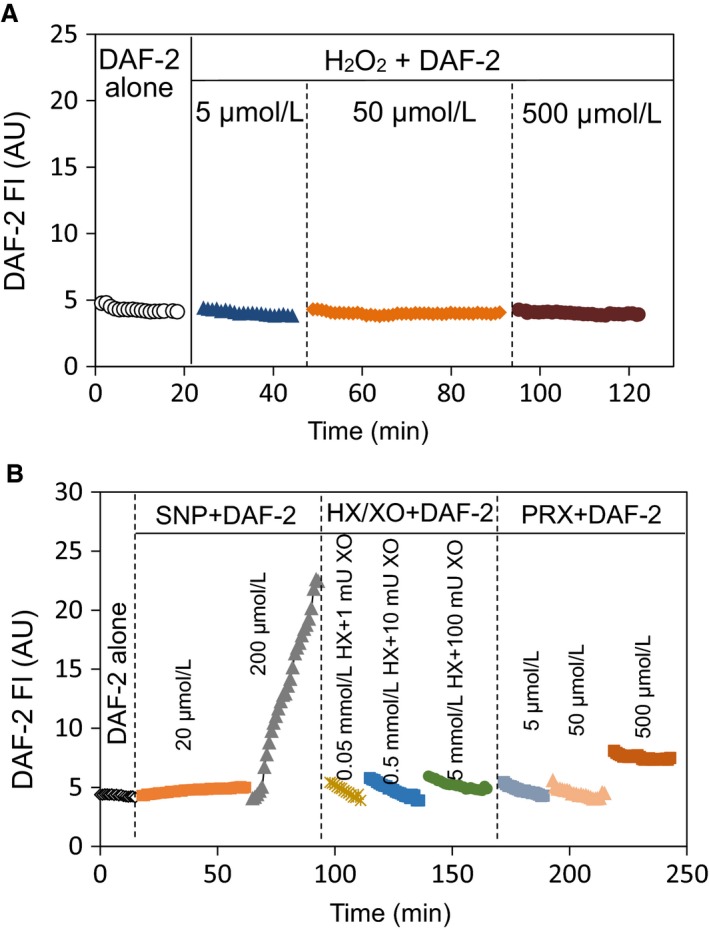
DAF‐2 specificity as NO indicator. To examine the specificity of DAF‐2 for NO, we conducted in vitro experiments to assess the direct reaction of DAF‐2 with H_2_O_2_, superoxide (generated by hypoxanthine/xanthine oxidase system, HX/XO), peroxynitrite (PRX), and NO donor sodium nitroprusside (SNP) at variable concentrations over 20‐min reaction time. Each reagent was individually added to BSA‐Ringer solution containing DAF‐2 (10 µmol/L), and the changes in DAF FI were recorded immediately using the same imaging system where NO was measured in intact microvessels. All FI measurements were acquired using identical settings. A and B show one set of measurements and triplet measurements were collected with each reagent. No significant changes in FI_DAF_ were found, except with NO donor, SNP at 200 µmol/L, and peroxynitrite at 500 µmol/L, suggesting that DAF‐2 does not directly interact with ROS and peroxynitrite in the absence of NO within the reagents’ concentrations we used in the experiments

Levels of endothelial NO were quantified at cellular level in individually perfused microvessels using a fluorescence imaging system and 4,5‐diaminofluorescein diacetate (DAF‐2 DA). The experimental rigs were the same as that used for Ca^2+^ measurements, except that a 12‐bit digital, cooled, charge‐coupled device camera (ORCA; Hamamatsu) was used for image acquisition. The excitation wavelength for DAF‐2 DA was selected by an interference filter (480/40 nm), and emission was separated by a dichroic mirror (505 nm) and a band‐pass barrier (535/50 nm). To minimize photo bleaching, a neutral density filter (0.5 N) was positioned in front of the interference filter and the exposure time was minimized to 0.12 sec at 1‐min intervals. All the images were acquired and analyzed using Metafluor software (Universal Imaging). During the experiment, each vessel was continuously perfused with albumin‐Ringer solution containing DAF‐2 DA (5 µmol/L). NO production was measured after the DAF‐2 loading reached the steady state (Zhou and He, 2011). Basal NO was measured for 10 min before perfusion with test reagents. All images were collected from a group of ECs located at the same focal plane of the vessel wall. At the end of the experiment, the NO donor, sodium nitroprusside (SNP, 50 mmol/L) was applied to the superfusate to ensure sufficient intracellular DAF‐2 to react with NO. Quantitative analysis was conducted at the individual EC level using manually selected regions of interest (ROIs) along the vessel wall. Each ROI covers the area of one individual EC as indicated by the fluorescence outline. The tissue autofluorescence was subtracted from all the measured FIs. The changes in FI_DAF_ upon addition of test reagents were expressed as the percentage of FI_DAF_ over the baseline FI_0_ (FI/FI_0_*100)_._ The rate of FI_DAF_ change was derived by the first differential conversion of cumulative FI_DAF_ over time. DAF‐2 specificity for NO detection was examined by mixing DAF‐2 (10 µmol/L) with albumin‐Ringer solution, H_2_O_2_ (5, 50 and 500 µmol/L), SNP (20 and 200 µmol/L), hypoxanthine/xanthine oxidase (0.05 mmol/L /1 mU, 0.5 mmol/L / 10 mU, and 5 mmol/L /100 mU), or peroxynitrite (5, 50, and 500 µmol/L) using the same experimental settings as those used for NO measurement on intact vessels. No significant increase in FI_DAF‐2_ was observed for all the test agents except SNP at 200 µmol/L that caused an accumulative increase in FI_DAF‐2_, indicating the specificity of DAF‐2 as a NO fluorescent indicator. Peroxynitrite at 500 µmol/L caused a small increase in FI_DAF_, but that concentration is beyond the range we used in the experiments. The representative measurements with each agent are shown in Fig 1.

### Confocal images of fluorescent immunostaining for nitrotyrosine and superoxide detection

Leica confocal system (SP2) was used to detect fluorescent immunostaining for nitrotyrosine and superoxide production with dihydroethidium (DHE) in H_2_O_2_‐perfused vessels. Images were acquired using Leica objective ×63 (HCX PL APO, NA 1.2) with ×1.5 electronic zoom, and the vertical step was 0.3 μm. The mean FI of each stack of ROIs that cover the vessel wall was quantified using Leica confocal software. The averaged FI of the perfused vessels was calculated. The ratio of the FI over control value was used to present the changes in the nitrated tyrosine content. The absolute mean FI in arbitrary unit from each vessel was calculated for DHE images to semi‐quantify the magnitude of superoxide production. Nitrotyrosine immunostaining was conducted in fixed vessels. The mesentery bearing each perfused vessel was fixed with paraformaldehyde, followed by 10‐min permeabilization with 0.1% Triton X‐100 and 1 h blocking with 5% bovine serum before overnight exposure to the mouse Anti‐Nitro tyrosine antibody (ab7048, abcam, USA) at 4°C on an automatic laboratory shaker. The tissue was then washed with BSA‐Ringer solution for three times before incubation with Alexa 488‐conjugated goat anti‐mouse secondary antibody (ab150113, abcam, USA) at room temperature for 3 h. For detection of superoxide, each vessel was loaded with DHE (10 µmol/L) for 30 min followed by perfusion with either BSA‐Ringer alone or containing H_2_O_2_ (10 µmol/L) for 30 min. The images were acquired with excitation at 488 nm and emission at 590–700 nm. To determine the source of superoxide, diphenylene iodonium (DPI, an NADPH oxidase inhibitor, 20 µmol/L) was applied to the vessel for 20 min after DHE loading, followed by perfusion with H_2_O_2_ (10 µmol/L) in the presence of DPI.

### Solutions and reagents

Mammalian Ringer solution (He et al., 1996) was used for dissecting mesenteries, superfusing tissues, and preparing perfusion solutions. The composition of the mammalian Ringer solution was (in mmol/L) 132 NaCl, 4.6 KCl, 2 CaCl_2_, 1.2 MgSO_4_, 5.5 glucose, 5.0 NaHCO_3_, 20 N‐2‐hydroxyethylpiperazine‐N’‐2‐ethanesulfonic acid (HEPES), and Na‐HEPES. All perfusates used for control and test perfusion contained BSA (10 mg/ml). Fura‐2 AM was purchased from Invitrogen. H_2_O_2_ (30%), DAF‐2, DAF‐2 DA, LaCl_3_, L‐NMMA, DPI, and DHE were purchased from Sigma. All the fluorescent dyes except for Alexa488‐conjugated secondary antibody (in BSA‐Ringer solution) were prepared in DMSO for stock solution, and at least 1:1000 dilution was made for the final working solutions. ONOO^−^ and degraded ONOO^−^ were purchased from EMD Millipore and were diluted in the BSA‐Ringer solution into final concentrations of 10 or 50 µmol/L. All perfusates containing the test reagents were freshly prepared before each cannulation.

### Data analysis and statistics

All values are presented as means ± SD. Each “*n*” represents the number of vessels (one vessel per rat). Paired t‐test was used for paired data analysis, that is, control and treatment were conducted in the same vessel. Unpaired *t*‐test with Welch's correction was used for data comparison between two groups, and one‐way or two‐way ANOVA with Tukey post hoc test was applied for multiple group comparison. A probability value of *P* < 0.05 was considered as statistically significant, and the exact or the range of p values is presented in figure legends.

## Results

### H_2_O_2_ induced NO‐dependent increases in microvessel Lp and EC [Ca^2+^]_i_

**Figure 2:**
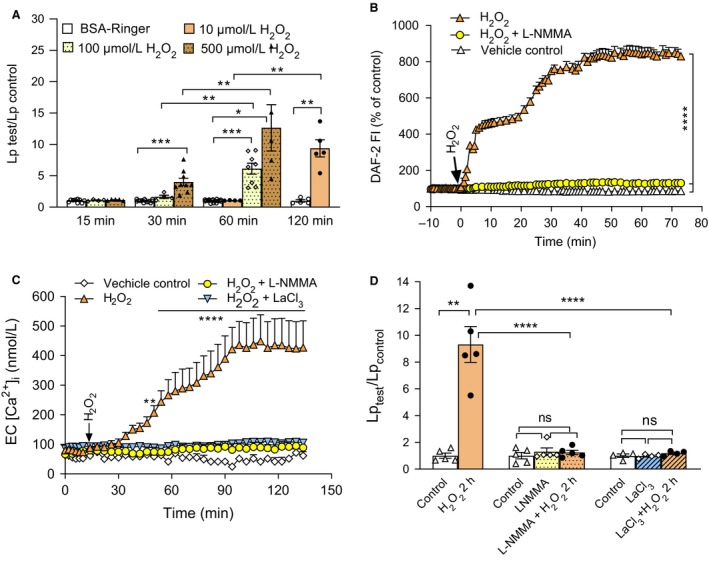
Causal relationship between H_2_O_2_‐induced eNOS‐derived NO production, increased EC [Ca^2+^]_i_, and microvessel Lp. (A) Summary results of H_2_O_2_‐induced time‐and dose‐dependent increases in microvessel Lp (*n* = 4–9 vessels per group). (B) H_2_O_2_ (10 µmol/L) perfusion induced a large amount of NO production that was inhibited by L‐NMMA (2 mmol/L, administered 30 min before H_2_O_2_ perfusion and present throughout the experiment). Ringer solution containing 1% BSA was used as vehicle control (*n* = 4 vessels per group). (C) H_2_O_2_ (10 µmol/L) perfusion induced delayed and progressive increases in EC [Ca^2+^]_i_. The application of L‐NMMA (2 mmol/L) or LaCl_3_ (50 µmol/L) before and during H_2_O_2_ perfusion prevented the H_2_O_2_‐induced increases in EC [Ca^2+^]_i_ (n = 4 per group). (D) L‐NMMA (2 mmol/L, *n* = 5) and LaCl_3_ (50 µmol/L, *n* = 4) prevented the H_2_O_2_‐induced delayed increases in microvessel Lp (Lp was measured after H_2_O_2_ perfusion for 2 h, *n* = 5). One‐way ANOVA with Tukey post hoc test were used for comparison between groups and paired t‐test was used for paired measurements conducted in the same vessel (A and D). Two‐way ANOVA with Tukey post hoc test were used for comparison of data between groups at the same time points (B and C), ****P < 0.0001. ***P < 0.001, **P < 0.005, *P < 0.05, ns *P ≥ *0.6

We previously demonstrated that H_2_O_2_ at pathologically relevant concentrations induced delayed and progressive increases in microvessel Lp, which was initiated by the activation of eNOS and excessive NO production (Zhou et al., 2009; Zhou et al., 2013). Fig 2A shows the summary results of H_2_O_2_‐induced time‐ and dose‐dependent increases in Lp. The H_2_O_2_‐induced endothelial NO production was measured in DAF‐2 DA‐loaded venules. Fig 2B shows the time course of H_2_O_2_‐induced NO production (presented as the mean changes in FI_DAF‐2_ pooled from four vessels). Since the FI_DAF_ increase in reaction to NO is cumulative (irreversible chemical conversion), the greater slope of the FI_DAF_ increase indicates a higher NO production rate and the plateau indicates no further increase in NO. When H_2_O_2_ (10 µmol/L) was perfused into the microvessel, there was an immediate increase in FI_DAF_ that reached 425 ± 18.1% of the baseline level in 5 min, followed by a lower sustained level. A second increase in FI_DAF_ was observed after 20 min of H_2_O_2_ perfusion with 280 ± 41% additional increase in FI_DAF_, and reached a plateau level at 41 min of H_2_O_2_ perfusion with a total of 830 ± 85% increase in FI_DAF_. In another four DAF‐2 DA‐loaded vessels, perfusion of albumin‐Ringer solution as a control showed approximately 1% increase per minute in FI_DAF_, representing basal NO production. Preperfusion of vessels with NOS inhibitor, L‐NMMA (2 mmol/L), for 30 min followed by the administration of H_2_O_2_ in the presence of L‐NMMA completely abolished H_2_O_2_‐induced NO production (*n* = 4, *P* < 0.0001). Then Lp was measured under the same experimental conditions that NO was measured. The application of L‐NMMA nearly abolished H_2_O_2_ (10 µmol/L)‐induced increases in microvessel Lp with decrease in mean Lp from 9.3 ± 1.3 to 1.3 ± 0.16 times of the control after 2‐h perfusion of H_2_O_2_ (Lp _test_/Lp _contro_, *n* = 5 per group, *P* < 0.0001, Fig. 2D).

The effect of H_2_O_2_ on EC [Ca^2+^]_i_ was examined in fura‐2 loaded microvessels (*n* = 5). The mean baseline EC [Ca^2+^]_i_ was 80 ± 10.5 nmol/L. Perfusion of venules with H_2_O_2_ (10 µmol/L) did not cause immediate increase in EC [Ca^2+^]_i_. A slow but progressive increase in EC [Ca^2+^]_i_ occurred after 15 min of H_2_O_2_ perfusion and reached a significant increase (207 ± 47.1 nmol/L) from control levels at 36 min of perfusion (p = 0.004), and plateaued at 436 ± 52.7 nmol/L with H_2_O_2_ perfusion for ~84 min (*P* < 0.0001 vs. vehicle control, Fig. 2C). The application of LaCl_3,_ a nonselective divalent cation channel blocker (50 µmol/L, added 20 min before H_2_O_2_ administration and present throughout the study), prevented H_2_O_2_‐induced increase in EC [Ca^2+^]_i_. The mean EC [Ca^2+^]_i_ before and after 2‐hour perfusion of H_2_O_2_ in the presence of LaCl_3_ were 80 ± 10.5 nmol/L and 96 ± 7.2 nmol/L, respectively (*n* = 4, *P* < 0.0001 vs. H_2_O_2_ perfusion alone, Fig. 2C), indicating Ca^2+^ influx to be the main source for H_2_O_2_‐induced increases in EC [Ca^2+^]_i_. Blocking Ca^2+^ influx by LaCl_3_ also prevented H_2_O_2_ (10 µmol/L)‐induced delayed increases in Lp (*n* = 4 & 5 per group, *P* < 0.0001, Fig. 2D), indicating the increased EC [Ca^2+^]_i_ after reaching certain threshold levels (>300 nmol/L, i.e., at least after 1h of H_2_O_2_ perfusion) to be responsible for increased Lp.

Since H_2_O_2_‐induced NO production is immediate and EC [Ca^2+^]_i_ did not increase until 15 min later, we predict that, if they are related, the delayed increase in EC [Ca^2+^]_i_ could be the result of the initial large amount of NO production in H_2_O_2_‐perfused vessels. To test this relationship, we measured EC [Ca^2+^]_i_ after blocking NO production using L‐NMMA in four vessels. L‐NMMA (2 mmol/L), without affecting the baseline EC [Ca^2+^]_i_ (67 ± 7.8 vs. 75 ± 10.8 nmol/L), completely abolished H_2_O_2_‐induced increase in EC [Ca^2+^]_i_ (Fig. 2C), indicating a causal relationship between H_2_O_2_‐induced excessive NO and the subsequent Ca^2+^ influx and eventually Ca^2+^ overload in ECs lining microvessel wall.

### H_2_O_2_ induced NO‐dependent tyrosine nitration in intact venules

**Figure 3:**
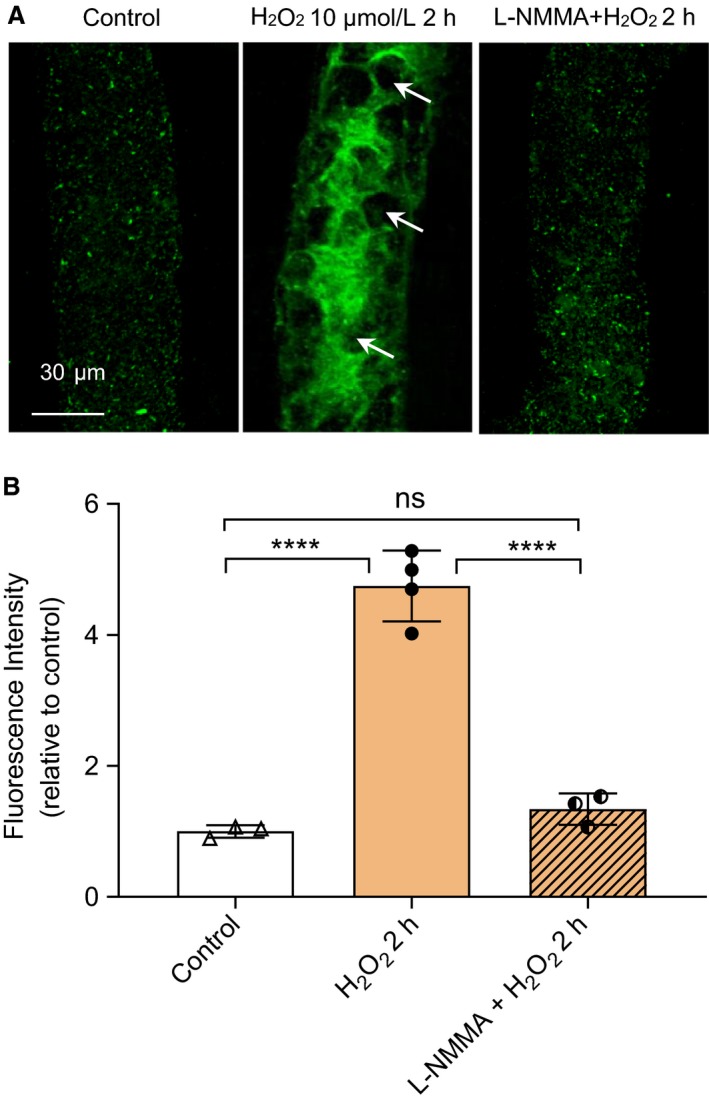
H_2_O_2_ induced NO‐dependent tyrosine nitration in microvessels. (A) Representative confocal images showing the fluorescent immunostaining of nitrotyrosine in vessels perfused with control (Ringer solution containing 1% BSA, left), H_2_O_2_ (10 µmol/L, middle), and H_2_O_2_ in the presence of L‐NMMA (2 mmol/L, right). (B) Summarized mean FI of nitrotyrosine staining in each group (*n* = 3‐4 per group, ****P < 0.0001, ns *P* = 0.55 (One‐way ANOVA with Tukey post hoc test)

To examine whether ONOO^−^ plays a role in H_2_O_2_‐induced increases in microvessel Lp, we examined nitrotyrosine, a biomarker of ONOO^−^, in H_2_O_2_‐perfused venules. In contrast to the low fluorescence signal in albumin‐Ringer‐perfused vessels (Fig. 3A left panel), H_2_O_2_ perfusion (10 µmol/L for 2 h) induced an extensive tyrosine nitration in the cytosol and cell membrane (Fig. 3A middle panel). Arrows indicate the nucleus of ECs without nitrated tyrosine formation. Importantly, L‐NMMA (2 mmol/L) significantly attenuated the nitrated tyrosine signal (Fig. 3A right panel. The fluorescence intensity (FI) was quantified in each vessel and summarized in Fig 3B. The mean FI in vessels with 2‐h H_2_O_2_ perfusion was 4.8 ± 0.27 times that of the control (*n* = 4, *P* < 0.0001), and the application of L‐NMMA before and during H_2_O_2_ perfusion (2 h) reduced the FI to 1.3 ± 0.1 times to that of the control (*n* = 3, *P* < 0.0001), indicating H_2_O_2_‐induced ONOO^−^ formation is NO dependent.

### H_2_O_2_ induced superoxide production in intact venules

**Figure 4:**
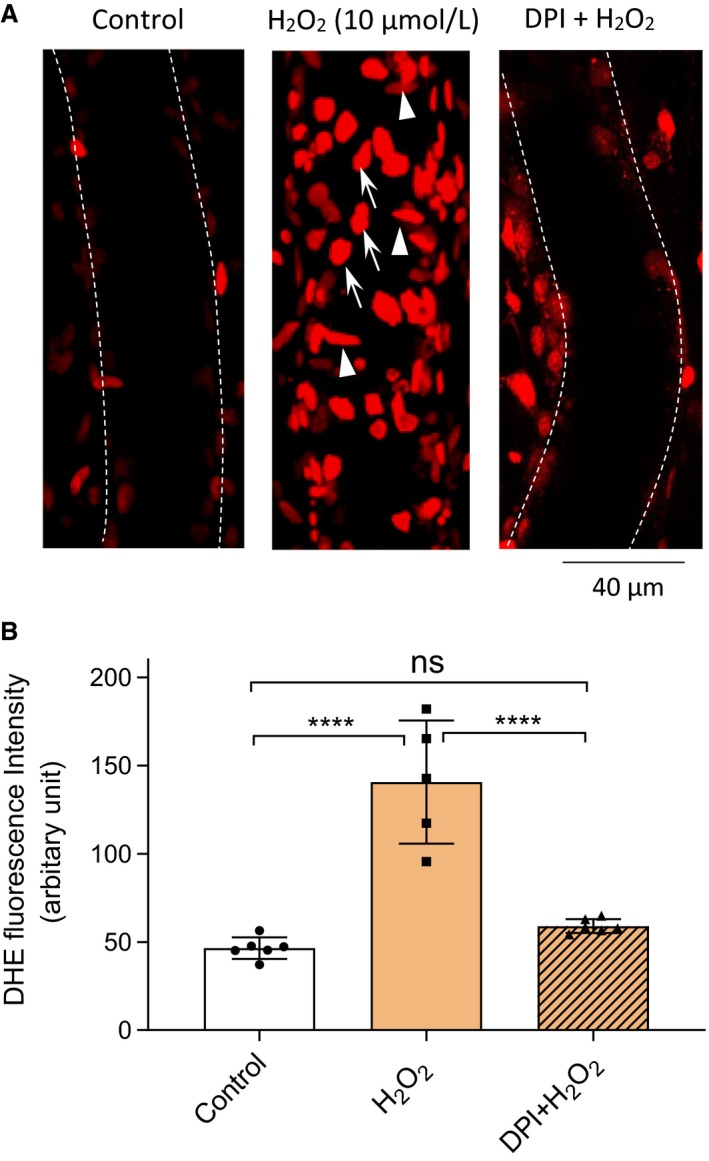
Superoxide production in H_2_O_2_‐perfused vessels. (A) Representative confocal images showing DHE staining is minimum in a control vessel (left panel), but markedly increased in the nuclei of both ECs (vertically orientated, indicated by arrows) and pericytes (horizontally orientated, indicated by arrow heads) after H_2_O_2_ (10 µmol/L) perfusion for 30 min (middle panel). Preperfusion of vessels with DPI (20 µmol/L) abolished H_2_O_2_‐induced superoxide production (right panel). Each image is the projection of the lower half of the vessel image stack with 0.5 µm per section. The dotted lines were used to outline the vessel walls. (B) Summarized mean FI of DHE in control (*n* = 6), H_2_O_2_ (*n* = 5) and H_2_O_2_ with DPI perfused vessels (*n* = 6). ****P < 0.0001, ns *P* = 0.52 (One‐way ANOVA with Tukey post hoc test)

NO reacts quickly with superoxide to form ONOO^−^. To examine the role of H_2_O_2_‐induced NO in the formation of ONOO^−^, we examined the intracellular superoxide levels in H_2_O_2_‐perfused vessels with DHE staining. As shown in Fig. 4A, a marked increase in the DHE FI was observed in both endothelium and pericytes after 30‐min perfusion of H_2_O_2_ (10 µmol/L), which was abolished by pretreatment of the vessels with an inhibitor of NADPH oxidase, diphenylene iodonium (DPI, 20 µmol/L,). The mean FIs of DHE were 48 ± 2.5, 141 ± 15.6, and 59 ± 0.5 AU in BSA‐Ringer (control, *n* = 6), H_2_O_2_ (*n* = 5), and H_2_O_2_ plus DPI‐perfused vessels (*n* = 6), respectively (Fig. 4B. *P* < 0.0001, H_2_O_2_ group vs. control and H_2_O_2_ plus DPI group, respectively).

### *Role of *ONOO^−^ *in H_2_O_2_‐induced increases in microvessel Lp and endothelial [Ca^2+^]_i_*

**Figure 5:**
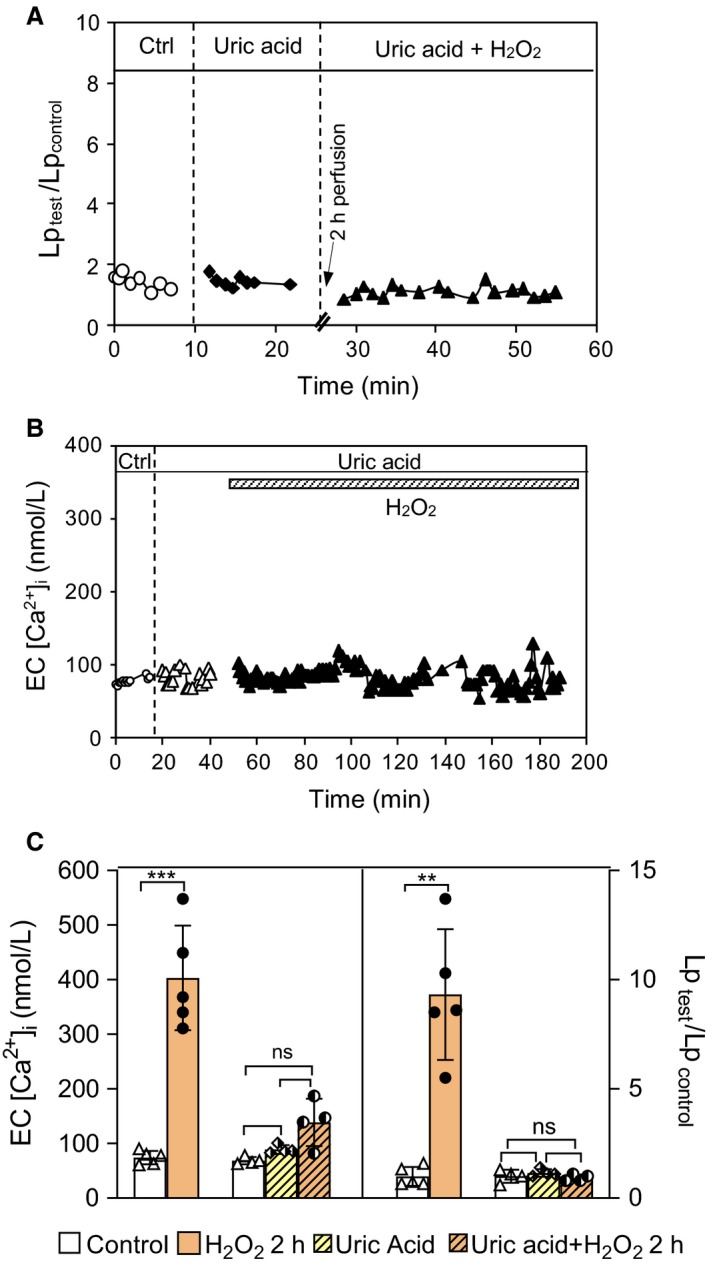
Role of ONOO^−^ in H_2_O_2_‐induced increases in EC [Ca^2+^]_i_ and microvessel Lp. (A and B) Two representative experiments showing that the application of uric acid (50 µmol/L), a ONOO^−^ scavenger, prevented H_2_O_2_‐induced increases in microvessel Lp and EC [Ca^2+^]_i_. Uric acid was added 20 min before and throughout the 2‐h H_2_O_2_ perfusion. C) Summarized results of EC [Ca^2+^]_i_ (left y axis) and Lp (right y axis). *n* = 4‐5 per group. ***P = 0.001, **P ≤ 0.003, ns *P* ≥ 0.6. (Paired *t*‐test or one‐way ANOVA were used for treatments conducted in the same vessel; and unpaired t‐test with Welch's correction was used for data comparison between two groups)

To examine whether NO‐derived ONOO^−^ accounts for the H_2_O_2_‐induced increases in EC [Ca^2+^]_i_ and microvessel Lp, the effects of H_2_O_2_ on EC [Ca^2+^]_i_ and microvessel Lp were investigated with the application of uric acid, a ONOO^−^ scavenger. Uric acid (50 µmol/L) perfusion for 20 min did not alter the baseline EC [Ca^2+^]_i_ (69 ± 4.7 vs. 85 ± 4.1 nmol/L) and Lp (2.3 ± 0.34 vs. 2.5 ± 0.19 × 10^‐7^ cm s^−1^ cmH_2_O^−1^, *P *> 0.8, *n* = 4 for each group), but significantly reduced H_2_O_2_‐induced increase in EC [Ca^2+^]_i_ from a mean peak value of 403 ± 43.0 (*n* = 5) to 139 ± 30.6 nmol/L (*n* = 4, *P* = 0.002), and completely prevented the H_2_O_2_‐induced increase in Lp (Lp_test_/ Lp_control_) from 9.3 ± 1.34 (*n* = 5) to 1.0 ± 0.08 times the control level (*n* = 4, *P* = 0.003). All values are compared after 2‐h perfusion of H_2_O_2_ (10 µmol/L). Fig. 5A,B show the representative experiments and Fig. 5C shows the results summary.

### Peroxynitrite induced delayed and progressive increases in microvessel Lp in a dose‐ and time‐dependent manner, a pattern similar to that observed in H_2_O_2_‐perfused vessels

**Figure 6:**
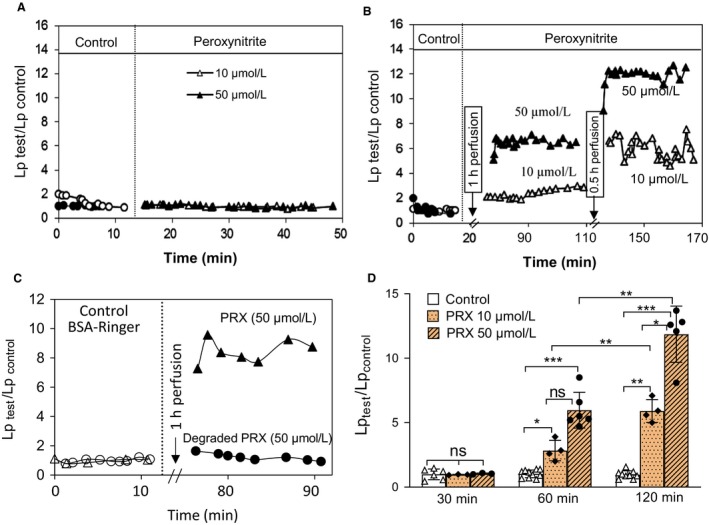
Peroxynitrite (ONOO^−^) induced dose‐ and time‐dependent increases in microvessel Lp. Representative experiments showing the changes in Lp when individual vessels were perfused with ONOO^−^ at 10 and 50 (µmol/L) for 30 min (A), 1 h, and 2 h respectively (B). (C) An overlay of two individual experiments showing that the 1‐h perfusion of ONOO^−^ (PRX) increased microvessel Lp, while the perfusion of the same concentration and time period of degraded PRX had no effect. (D) Summary of PRX‐induced time‐ and dose‐dependent increases in microvessel Lp. *P < 0.03, **P < 0.003, ***P < 0.0006, ns *P* > 0.9, ns*P = 0.054

To investigate the direct effect of ONOO^−^ on microvessel permeability, we perfused ONOO^−^ to microvessels and measured the changes in Lp. The mean baseline Lp was 2.1 ± 0.16 × 10^−7^ cm s^−1^ cmH_2_O^−1^ (*n* = 25). No changes in Lp were observed with ONOO^−^ perfusion for 30 min at 10 and 50 µmol/L (Lp_test_/Lp_control_ was 1.0 ± 0.02 and 1.0 ± 0.03, respectively, *n* = 3 per group, Fig. 6A,D). However, extending the perfusion duration to 1 and 2 h resulted in time‐ and dose‐dependent Lp increases similar to that observed in H_2_O_2_‐perfused vessels (Fig 2A). Lp increased significantly to 2.8 ± 0.45 (*P* = 0.03) and 5.9 ± 0.44 (*P* = 0.003) times the control at 10 µmol/L, and to 6.0 ± 0.57 and 12.0 ± 0.96 times the control at 50 µmol/L (*P* < 0.001) after 1 and 2 h of ONOO^−^ perfusion, respectively (Fig. 6B,D, *n* = 4‐6 per group). We also examined the effect of degraded peroxynitrite on microvessel Lp in two vessels. No changes in Lp were observed after 1‐h perfusion of degraded peroxynitrite (50 µmol/L). Fig 6C represents the contrast of the Lp changes in peroxynitrite‐ and degraded peroxynitrite‐perfused vessels.

### Peroxynitrite‐induced progressive increases in EC [Ca^2+^]_i_ contributed to the increases in microvessel Lp

**Figure 7:**
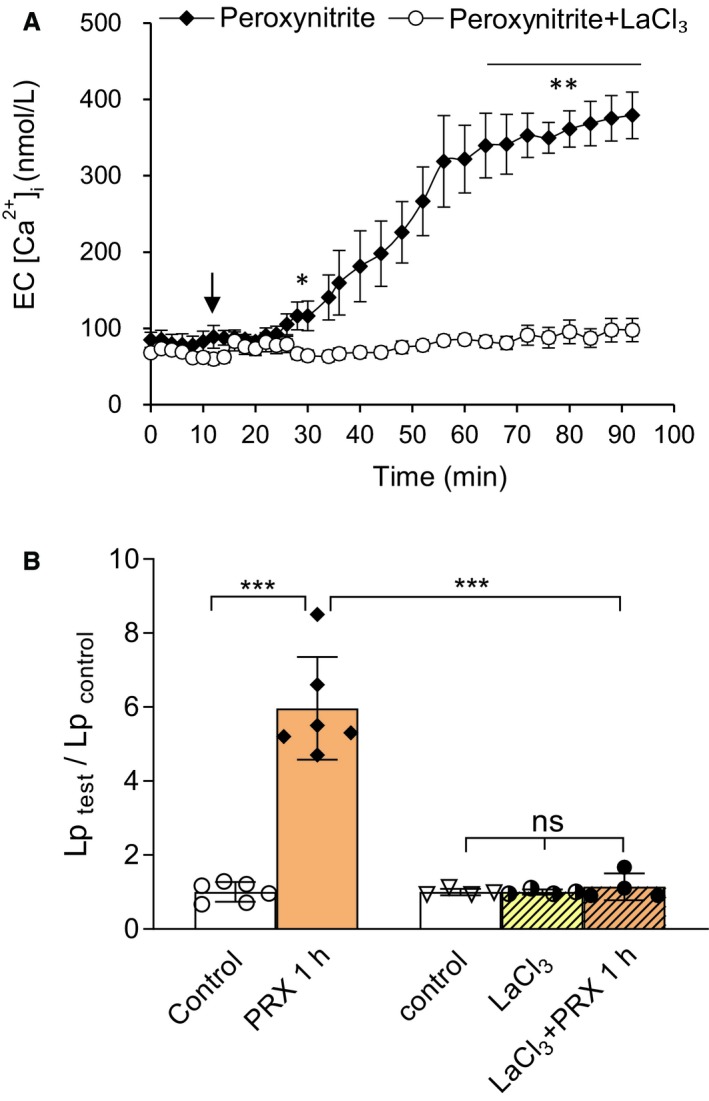
ONOO^−^‐induced Ca^2+^ influx contributes to increased microvessel Lp. (A) The time course of the changes in EC [Ca^2+^]_i_ when vessels were perfused with ONOO^−^ (50 µmol/L) in the absence (*n* = 4) or presence of LaCl_3_ (50 µmol/L, N = 3). Perfusion of ONOO^−^ induced delayed and progressive increases in EC [Ca^2+^]_i_ and the application of LaCl_3_ that blocked Ca^2+^ influx prevented such effect. Arrow indicates the initiation of ONOO^−^ perfusion. (B) Summarized results of ONOO^−^‐induced Lp increase and its dependency on Ca^2+^ influx demonstrated by the effect of LaCl_3_ on ONOO^−^‐perfused vessels. *P < 0.05, **P = 0.0027, ***P < 0.0005, ns *P* = 0.6

The causal relationship between ONOO^−^ and EC [Ca^2+^]_i_ was further examined in seven vessels loaded with fura‐2. Baseline EC [Ca^2+^]_i_ was 82 ± 12.0 nmol/L. A significant increase in EC [Ca^2+^]_i_ occurred at 20 ± 4.0 min of ONOO^−^ perfusion (50 µmol/L), and progressively reached to a sustained level of 375 ± 30.0 nmol/L at 78 ± 2.3 min of ONOO^−^ perfusion (*n* = 4, Fig. 7A). No changes in EC [Ca^2+^]_i_ were observed on vessels perfused with albumin‐Ringer for 1 h (n = 3, data not shown). Preperfusion of vessels with LaCl_3_ (50 µmol/L) for 20 min did not affect baseline EC [Ca^2+^]_i_, but prevented ONOO^−^‐induced increases in EC [Ca^2+^]_i_ (the mean EC [Ca^2+^]_i_ was 98 ± 15.2 nmol/L at 80 min of ONOO^−^ perfusion (*n* = 3, *P* = 0.0027, Fig. 7A), indicating the role of Ca^2+^ influx in ONOO^−^‐induced increase in EC [Ca^2+^]_i_.

The role of Ca^2+^ influx in ONOO^−^‐induced Lp increase was then examined. The application of LaCl_3_ prevented the increased Lp in ONOO^−^ (50 µmol/L, 1 h)‐perfused vessesls. The Lp_test_/Lp_control_ reduced from 6.0 ± 0.57 (*n* = 6) to 1.1 ± 0.18 times the control (*n* = 4, *P* = 0.0002, Fig. 7B), indicating that the sustained increase in EC [Ca^2+^]_i_ to be responsible for ONOO^−^‐induced Lp increase.

### Peroxynitrite induced eNOS activation and NO production and Ca^2+^ influx dependency

**Figure 8:**
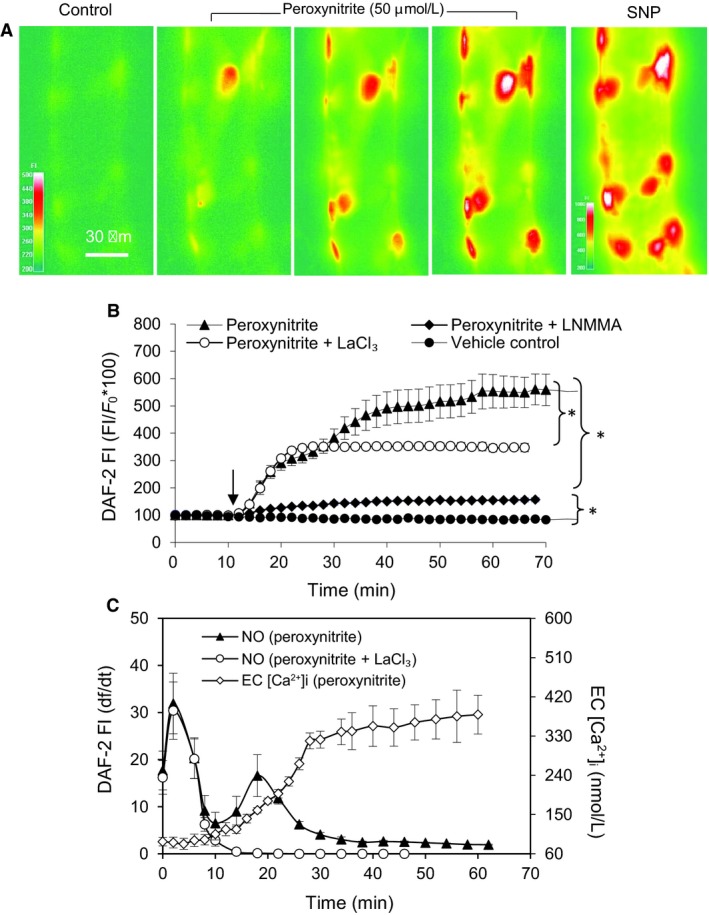
ONOO^−^ induced Ca^2+^influx‐independent and ‐dependent eNOS activation and NO production in perfused venules. (A) Representative DAF‐2 fluorescent images in one individual vessel showing the changes in FI_DAF‐2_ at selected time points upon ONOO^−^ (50 µmol/L) perfusion. Sodium nitroprusside (SNP, 50 mmol/L) was applied to the superfusate at the end of the experiment to ensure sufficiently loaded DAF‐2 to react with NO. Scale bar indicates the FI_DAF‐2_ in arbitrary unit. (B) Pooled cumulative FI_DAF‐2_ curve in ONOO^−^‐perfused vessels in the absence (*n* = 4) and presence of L‐NMMA (2 mmol/L, *n* = 4) or LaCl_3_ (50 µmol/L, *n* = 4). Ringer solution containing 1% BSA was applied as vehicle control (*n* = 3). C) ONOO^−^‐induced changes in NO production rate (df/dt, left Y axis) superimposed with the changes in EC [Ca^2+^]_i_ (right Y axis). *P = 0.015 (Two‐way ANOVA)

To evaluate the relationship between ONOO^−^ and eNOS activity, NO was measured in ONOO^−^ (50 µmol/L)‐perfused vessels (n = 4). Perfusion of vessels with ONOO^−^ induced an immediate increase in NO production with an initial peak rate at 31.9 ± 6.43% min^‐1^ for 2 min followed by a rate of 6.5 ± 2.30% min^‐1^ till 10 ± 1.4 min (Fig. 8C). This initial increase in FI_DAF_ (ΔFI%) was 332 ± 25% of baseline control. With continuous ONOO^−^ perfusion, a second peak of NO production occurred at ~18 min with a rate of 16.6 ± 4.50% min^‐1^ and fell to 1.9 ± 0.10% min^−1^ at ~ 62 min (Fig. 8C). This second phase of FI increase (ΔFI%) was 222.0 ± 18.50% of the control (Fig. 8A,B). Control experiments in vessels perfused with albumin‐Ringer solution (*n* = 3) only showed basal NO production at a rate of 0.1%/min (Fig. 8A,B).

By comparing ONOO^−^‐induced NO production with the time course of the increases in EC [Ca^2+^]_i_, we found that the initial increase in NO production occurred in the absence of increased EC [Ca^2+^]_i_, whereas the second phase of NO production occurred concomitantly with increases in EC [Ca^2+^]_i_ (Fig. 8C). To examine the role of Ca^2+^ influx in ONOO^−^‐induced NO production, NO was measured on four vessels pretreated with LaCl_3_ (50 µmol/L). As described above (Fig. 7A), LaCl_3_ prevented ONOO^−^‐induced increases in EC [Ca^2+^]_i_. Under the same experimental conditions as those EC [Ca^2+^] were measured, NO measurements showed that inhibition of Ca^2+^ influx by _3_ had no effect on the ONOO‐induced initial NO production, but attenuated the second phase of NO production (ΔFI% reduced from 222.0 ± 18.50% to 15.0 ± 3.25% of control, Fig. 7B,C). These data suggest that ONOO^−^‐induced initial NO does not require increased Ca^2+^ influx, but the second phase of NO production was initiated by the increased EC [Ca^2+^]_i_.

We also examined whether ONOO^−^‐induced NO production was a result of NOS activation. Treatment of vessels with NOS inhibitor, L‐NMMA (2 mmol/L), 20 min before and during ONOO^−^ perfusion significantly decreased ONOO^−^‐induced NO production. FI_DAF_ reduced from 570 ± 141.0% to 154 ± 50.0 % of control, (*n* = 4, Fig. 8B).

## Discussion

**Figure 9:**
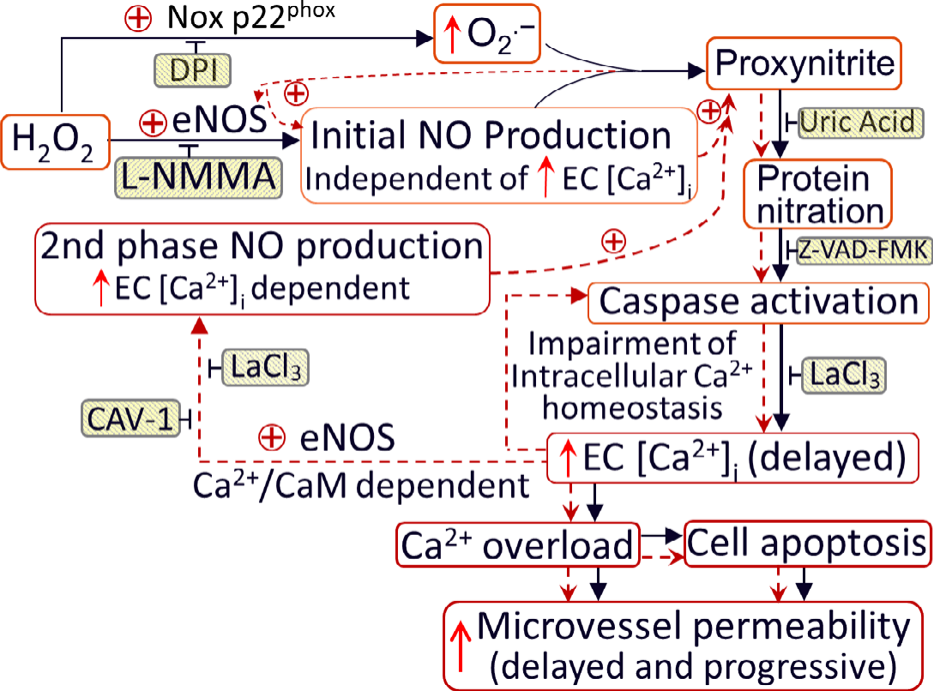
Schematic diagram illustrating the mechanisms of H_2_O_2_‐induced microvessel barrier dysfunction. H_2_O_2_ at pathophysiological levels activate eNOS and NADPH oxidase in the endothelium and pericytes, leading to increased formation of NO and O_2_^−^. The temporal and spatial proximity of largely produced NO and O_2_^−^ promote the formation of ONOO^−^, a more potent oxidant, leading to ONOO^−^‐mediated lipid peroxidation, protein tyrosine nitration, subsequent impairment of intracellular Ca^2+^ homeostasis, and vascular cell apoptosis. Both NO‐derived ONOO^−^ and the delayed increases in EC [Ca^2+^]_i_ are able to further activate eNOS, resulting in amplified NO production and NO‐dependent downstream cascade. These positive feedback loops, indicated by red dotted lines, play an important role in sustained production of ONOO^−^, and eventually ONOO^−^‐mediated endothelial cell Ca^2+^ overload, vascular cell apoptosis, and progressively increased microvessel permeability. The application of inhibitors (shaded boxes) blocks the downstream events. This diagram include both previous (Zhou et al., 2009; Zhou et al., 2013) and current studies. DPI (diphenylene iodonium, NADPH oxidase inhibitor); eNOS: endothelial nitric oxide synthase; CAV‐1: Caveolin‐1 (eNOS inhibitor); z‐VAD‐FMK: caspase inhibitor

Our study, using quantitative measurement of microvessel permeability in combination with fluorescence and confocal microscopy, identified a novel mechanism by which H_2_O_2_, a commonly known reactive oxygen species essential for the pathogeneses of cardiovascular diseases, interplays with superoxide, eNOS‐derived NO, and ONOO^−^, leading to EC Ca^2+^ overload, vascular cell damage, and barrier dysfunction in intact vessels. H_2_O_2_ at a pathophysiological relevant concentration (10 µmol/L) (Lacy et al., 2000; Banerjee et al., 2003) induced eNOS activation with a large amount of NO production. Instead of causing an immediate increase in microvessel permeability that occurred in PAF or other inflammatory mediator‐stimulated microvessels (He et al., 1996; Zhu and He, 2005; Zhou and He, 2010), this large amount of NO (~10 times the amount of NO detected in PAF‐perfused microvessels) was shown to be responsible for intracellular Ca^2+^ accumulation, subsequent cell apoptosis, and the delayed and progressive increases in microvessel permeability ( Zhou et al., 2013). Concomitant with the excessive NO production, a marked production of superoxide, as well as extensive protein tyrosine nitration, a biomarker for ONOO^−^, were detected in H_2_O_2_‐perfused vessels, indicating H_2_O_2_‐induced ONOO^−^ formation in intact microvessels. Functionally, H_2_O_2_‐induced increases in microvessel Lp was prevented by pretreatment of the vessels with uric acid, a strong ONOO^−^ scavenger, suggesting a causal relationship between ONOO^−^ and H_2_O_2_‐induced microvessel barrier dysfunction. The direct evidence of the role of ONOO^−^ in H_2_O_2_‐induced vascular barrier dysfunction was demonstrated by exogenously applied peroxynitrite to the microvessels, which showed a dose‐ and time‐dependent increase in microvessel Lp, a pattern similar to that observed in H_2_O_2_‐perfused vessels. Importantly, ONOO^−^, a downstream product derived from NO, is demonstrated to cause further eNOS activation and NO production. This reciprocal interaction between H_2_O_2_, ONOO^−^_,_ and eNOS constitutes a self‐promoted amplification of ONOO^−^. Taken together, this study provides the first in vivo evidence that ONOO^−^, derived from H_2_O_2_‐induced NO and superoxide, further activates eNOS, leading to a self‐promoted amplification of signaling cascades, resulting in H_2_O_2_‐induced ONOO^−^‐mediated cell damage and microvessel barrier dysfunction. Details are illustrated in Fig. 9.

### Role of eNOS‐derived NO production in the regulation of H_2_O_2_‐induced microvessel barrier dysfunction

Previous studies from our laboratory and others have demonstrated that eNOS‐derived excessive NO plays an essential role in inflammatory mediators‐induced vascular leakage (Mayhan, 1992; Yuan et al., 1993; He et al., 1997; Hatakeyama et al., 2006; Zhou and He, 2010; Zhou and He, 2011). A typical inflammatory mediator‐induced increase in microvessel permeability usually is immediate and transient, which is associated with the transient formation of endothelial gaps between adjacent ECs (Majno and Palade, 1961; Wu and Baldwin, 1992; Baluk et al., 1997; Jiang et al., 2008; Zhou and He, 2011). In contrast to the inflammatory mediator‐induced immediate and transient increase in microvessel permeability, H_2_O_2_ caused a delayed and progressive increase in microvessel Lp in a dose‐ and time‐dependent manner (Zhou et al., 2009; Zhou et al., 2013), indicating the involvement of different mechanisms. Our study demonstrated that H_2_O_2_ at pathophysiologically relevant concentrations (10 µmol/L) induce both Ca^2+^‐independent and ‐dependent eNOS activation and the excessively produced NO triggers caspase activation and the impairment of intracellular Ca^2+^ homeostasis, leading to vascular cell apoptosis and consequently delayed and progressive increases in microvessel permeability ( Zhou et al., 2013). In this case, the H_2_O_2_‐induced initial NO production in the absence of increased EC [Ca^2+^]i, although being five times more than that observed in PAF‐perfused vessels, did not cause immediate increase in microvessel permeability (Zhu and He, 2005; Zhou and He, 2010). These findings suggest that the excessive NO‐mediated vascular barrier dysfunction may depend on the activation of other concurrent signaling pathways, such as increased EC [Ca^2+^]_i_, or the location of NO production (Sanchez et al., 2006), thus the pattern of NO‐mediated increase in microvessel permeability is stimulus specific.

Our previous study illustrated that the H_2_O_2_‐induced initial phase of eNOS activation is predominantly through increased phosphorylation at Ser^1177^ independent of elevated EC [Ca^2+^]_i_, whereas the second phase of eNOS activation is mainly triggered by increased EC [Ca^2+^]_i_ and Ca^2+^/CaM binding (inhibited by caveolin‐1, CAV‐1), coordinated with decreased phosphorylation at Thr^495^ (Zhou et al., 2013). Thus, not only does the delayed increases in EC [Ca^2+^]_i_ contribute to EC Ca^2+^ overload, it also triggers a second phase of Ca^2+^‐dependent NO production, further amplifying the NO‐dependent signaling cascade. This notion is supported by the fact that blocking NOS activation by L‐NMMA prevented the delayed and progressive increases in EC [Ca^2+^]_i_ (Fig. 2C). The sustained increase in EC [Ca^2+^]_i_, an indication of impaired Ca^2+^ homeostasis, appears to be essential for vascular cell apoptosis and subsequently increased microvessel permeability.

### *Role of NO‐derived *ONOO^−^ *in H_2_O_2_‐induced increases in microvessel Lp*

Although NO has been reported to have potential cytotoxic effects, many of them are attributed to its oxidation product, particularly ONOO^−^ rather than NO itself (Xia et al., 1996; Riobo et al., 2001; Pacher et al., 2007). NO reacts rapidly with superoxide to form ONOO^−^, a strong oxidant, capable of oxidizing and nitrating lipid, protein, and DNA, resulting in cell death and tissue injury (Radi et al., 1991; Pacher et al., 2007; Szabo et al., 2007). Consistent with this concept, our results support that H_2_O_2_‐induced excessive NO‐derived ONOO^−^ is the key contributing to increased microvesel permeability in intact rat venules. This conclusion is supported by the following evidence: (1) H_2_O_2_ induced concomitant NO and superoxide production, the two elements required to form ONOO^−^, (2) H_2_O_2_ induced NO‐dependent extensive protein tyrosine nitration (a commonly recognized biomarker for ONOO^−^) in the vascular wall, (3) uric acid, an endogenous ONOO^−^ scavenger (Ames et al., 1981; Hooper et al., 1998; Tsukada et al., 2000; Walford et al., 2004; Kuzkaya et al., 2005), prevented H_2_O_2_‐induced impairment of Ca^2+^ homeostasis and increases in microvessel permeability; and (4) exogenously applied ONOO^−^ induced a delayed and progressive increases in EC [Ca^2+^] and microvessel Lp in a dose‐ and time‐dependent manner, a pattern similar to that observed in H_2_O_2_‐perfused vessels.

The H_2_O_2_‐induced superoxide production has been considered as an important feed‐forward mechanism for ROS‐mediated pathogenesis of cardiovascular diseases (Cai, 2005). In vitro studies showed that inhibition of p22^phox^ by siRNA and knockout of gp91^phox^ (the two transmembrane subunits of NAD(P)H oxidase) prevented the H_2_O_2_‐induced increases in superoxide production in vascular endothelial cells (Li et al., 2001; Djordjevic et al., 2005). Using confocal microscopy, we detected a ~threefold increase in DHE FI in the nuclei of both ECs and pericytes as early as 30 min of H_2_O_2_ perfusion, indicating a large amount of superoxide production (Fig. 4). Within the same period, NO production increased ~eightfold (indicated by the increased FI_DAF‐2_, Fig. 2B). When both NO and superoxide are produced in close proximity, a spontaneous ONOO^−^ formation is expected in the H_2_O_2_‐perfused vessels. Our results that DPI, a commonly used NAD(P)H oxidase inhibitor, prevented the increases in superoxide production in both endothelium and pericytes in H_2_O_2_‐perfused intact vessels (Fig. 4) support NAD(P)H oxidase as the primary source for the H_2_O_2_‐induced superoxide production.

More direct evidence of H_2_O_2_‐induced ONOO^−^ formation is the extensive NO‐dependent tyrosine nitration detected after 2‐h H_2_O_2_ perfusion (Fig. 3). Nitrotyrosine has been recognized as an important biomarker for ONOO^−^ and an indication of systemic nitroxidative stress (Prutz et al., 1985; Reiter et al., 2000; Radi, 2004; Radi, 2013).

The application of uric acid, an endogenous ONOO^−^ scavenger (Hooper et al., 1998; Tsukada et al., 2000; Walford et al., 2004; Kuzkaya et al., 2005), that abolished the H_2_O_2_‐induced increases in EC [Ca^2+^]_i_ (Fig. 5B,C) and microvessel Lp (Fig. 5A,C) further support a role of ONOO^−^ in H_2_O_2_‐mediated changes in microvessels. Furthermore, the direct perfusion of ONOO^−^ in microvessels showed delayed and progressive increases in both EC [Ca^2+^]_i_ and microvessel Lp in a dose‐ and time‐dependent manner (Fig. 6), a similar pattern to that observed in H_2_O_2_‐perfused vessels (Zhou et al., 2009; Zhou et al., 2013), and inhibition of Ca^2+^ influx with LaCl_3_ prevented Ca^2+^ accumulation in ECs and the increased Lp in peroxynitrite‐perfused vessels (Fig. 7). These results demonstrate a direct role of ONOO^−^ in the impairment of Ca^2+^ homeostasis and vascular barrier dysfunction.

### *Interaction between *ONOO^−^ *and eNOS‐derived NO production: a self‐promoted amplification, contributing to *ONOO^−^ *‐mediated vascular damage and barrier dysfunction*

Currently, the role of ONOO^−^ in eNOS activity, particularly under in vivo conditions, remains unknown. While some early studies reported that ONOO^−^ induced coronary vasodilation in isolated rat hearts and coronary arterial rings of dogs through increased NO production (Liu et al., 1994; Villa et al., 1994), other studies on cultured bovine ECs indicated that ONOO^−^‐induced eNOS phosphorylation at Ser^1179^ (corresponding to Ser^1176^ in human and rats) increased O_2_^‐.^production, rather than that of NO (Zou et al., 2002; Zou et al., 2002). Using quantitative fluorescent imaging in intact venules, our study demonstrated that ONOO^−^ induced a large amount of NO that was abolished by L‐NMMA, indicating eNOS‐derived NO production (Fig. 7). Similar to what have been observed in H_2_O_2_‐perfused vessels (Shen et al., 1998), ONOO^−^ induced two phases of eNOS‐derived NO production. As shown in Fig. 8C, the initial NO production with a higher peak rate occurred in the absence of increases in EC [Ca^2+^]_i_, whereas the second phase NO production is slow and concomitant with progressively increased EC [Ca^2+^]_i_. Blockade of Ca^2+^ influx by LaCl_3_ did not affect the initial NO production, but abolished the NO production in the second phase (Fig8B,C), suggesting that peroxynitrite‐induced initial eNOS activation is independent on Ca^2+^ increase, whereas the second phase of eNOS activation is Ca^2+^ dependent. This interesting finding suggests that ONOO^−^, a downstream product formed by NO and superoxide, further amplified NO production, resulting in an amplification of NO‐dependent signaling and augmented ONOO^−^‐mediated vascular cell damage and barrier dysfunction.

### Methodology assessment

Vascular barrier function is usually referred to vascular permeability to both fluid and solutes. In this study, microvascular permeability is determined by measuring hydraulic conductivity, Lp, one of the three permeability coefficients that assesses the transport of fluid. Although microvessel permeability to different sizes of solute was not measured, experimental evidence showed that Lp well correlates with the transports of small‐ to intermediate‐sized molecules (Michel and Curry, 1999). Importantly, most of the increases in solute flux (including large molecules) are coupled to water flows. When permeability increases, the magnitude changes in fluid or small molecules could be different from macromolecules, depending on transport pathways involved, they usually followed the same time course as the changes in Lp (Rutledge et al., 1990; Rutledge et al., 1995; Michel and Curry, 1999). The permeability coefficients are more quantitative and accurate measures of vascular wall properties than the commonly used flux or leaky sites, since they are not affected by unknown variations of hemodynamic factors and surface area for transport (Michel and Curry, 1999). Importantly, all the studies presented here were conducted in individually perfused intact microvessels under well‐controlled conditions, and the changes in NO, superoxide, ONOO^−^, and EC [Ca^2+^]_i_ were all measured under the same experimental conditions similar to those where Lp was measured, which enabled us to directly correlate the changes in signaling pathways with microvessel permeability.

Another advantage of using individually perfused vessel to investigate the effects of reactive reagents is that the solution containing H_2_O_2_ or ONOO^−^ in the perfusion pipette constantly refreshes the perfusate in the vessel lumen, which provides a relatively constant concentration of H_2_O_2_ or ONOO^−^ to the cells lining vascular walls. This constantly refreshed perfusate makes a fundamental difference from the one time addition of reactive reagents to the tissue or cell culture medium that results in an immediate reaction of the reactive agents with the cells and other components in the medium and lost it activity quickly. In those studies, usually hundreds of µmol/L or mmol/L concentrations of peroxide (far more than the pathological concentrations detected in the plasma) were used in order to show some of the responses (Alexander et al., 2000; Kevil et al., 2000; Lee et al., 2006; Jin et al., 2012; Suresh et al., 2015). We also knew that H_2_O_2_ or ONOO^−^ even reacts with marker cells (about 1% RBCs) in the perfusate and affects the effectiveness of the treatment. That is why we perfuse the vessel without marker cells for a certain time period and only add the marker cells during Lp measurements. This also explains why we can get the responses with pathological range of plasma H_2_O_2_ concentrations (10 µmol/L). Based on our current (Figs 2, 6, 7) and previous studies (He et al., 1996; Zhou et al., 2013), the cell marker‐free perfusate in the presence of 1% BSA is able to well maintain the baseline Lp, EC [Ca^2+^]_i_ and basal NO up to 2‐h perfusion period which we have tested.

Compared to normal blood, our perfused vessels lack blood cells, plasma SOD, catalase, and substances released from blood cells, which could affect the production of ROS, reactive nitrogen species (RNS), and NO. Under diseased conditions, measured plasma peroxide levels are close to 10 µmol/L (Lacy et al., 2000). We consider that the detected plasma H_2_O_2_ level is the effective level of peroxide that affects endothelial cells lining the vascular walls and is the net balance of the constant production of ROS and its reaction with plasma enzymes such as SOD and catalase in the vasculature. Our experimental conditions bypassed the constant ROS production and catalytic processes when blood is circulating in the vasculature, and applied this pathologically relevant net plasma H_2_O_2_ concentration to the vessel lumen, which allows us to investigate its direct effect on vascular walls and avoid other confounding factors.

The use of DHE to detect intracellular formation of superoxide has been assessed and characterized by many studies. The formation of 2‐hydroxyethidium is considered as the specific reaction of DHE with superoxide and not with other biologically relevant oxidants, which can be separated from its parent DHE and ethidium (Zielonka et al., 2008), including vessel segment studies and confirmed with high performance liquid chromatography (HPLC, Dikalov et al. (2007)). The advantage of using the DHE fluorescence imaging in our perfused intact vessels is its ability to provide spatial detection of superoxide production at vascular walls using the same experimental approach where other signaling changes and microvessel permeability are measured, which could not be achieved with other methods. The pharmacological inhibitors used in this study such as DPI and uric acid showed consistent results as NADPH oxidase inhibitor and ONOO^−^ scavenger (Hooper et al., 1998; Kean et al., 2000; Kim et al., 2018; Pietruczuk et al., 2019), respectively, even though they may have nonspecific effects. These additional supporting evidence, together with the results of DHE staining and the nitrotyrosine immunostaining provided multiple lines of evidence to support the H_2_O_2_‐induced superoxide production and subsequent ONOO^−^ formation.

One potential concern is the specificity of using DAF‐2 to measure NO in the presence of the reactive oxidants (Jourd'heuil, 2002). DAF‐2 DA has been used to measure intracellular NO in a wide range of biological systems. However, since the interaction of DAF‐2 with NO to form DAF‐2 triazole form (DAF‐2T, high fluorescence product) requires the presence of O_2_, it raises concerns about the potential effects of oxidants on DAF‐2T conversion (Jourd'heuil, 2002; Wardman, 2007; Hall and Garthwaite, 2009). To address this issue, we have carefully characterized this NO probe in individually perfused vessels and demonstrated the cautious procedures to avoid non‐NO‐related DAF‐2 FI (Zhou and He, 2011). We also assess the specificity of DAF‐2 by measuring its direct reaction with H_2_O_2_, superoxide (generated by HX/XO), and ONOO^−^ in solution using the same imaging system when NO was measured in intact microvessels. The results shown in Fig 1 demonstrated that DAF‐2 (10 µmol/L, closer to intracellular DAF‐2 concentration when 5 µmol/L DAF‐2 DA was used to load vessels) does not directly interact with ROS and ONOO^−^ in the absence of NO within the reagents' concentrations we used in the experiments, although at this point we may not exclude the possibility that the presence of oxidants may facilitate the DAF‐2T formation with increased flux of NO and enhance DAF‐2 FI. Our results show that the use of NOS inhibitor completely blocked the H_2_O_2_‐ or ONOO^−^‐induced increases in DAF‐2 FI indicating a strong association between DAF‐2T and increased NO production. We consider that DAF‐2 is the most reliable specific NO indicator comparing with other up to date methods. Since the H_2_O_2_‐ and ONOO^−^‐induced DAF‐2 FI changes are in large magnitude, some contributions from oxidant effect will not change the nature of our findings. Especially, our previous studies have provided the validation of the NO measurements with DAF‐2 by conducting parallel studies with immunostaining of eNOS activation (Zhou et al., 2013).

In conclusion, our study uncovered a novel mechanism of elevated H_2_O_2_‐mediated microvascular dysfunction that commonly occurs in many cardiovascular diseases. H_2_O_2_ at pathophysiologically relevant concentrations, induces a large amount of eNOS‐derived NO concomitant with increased superoxide production, and subsequent formation of ONOO^−^, a more potent reactive nitrogen species (RNS). The formed ONOO^−^ is able to further activate eNOS and augment NO production, resulting in a self‐promoted augmentation of nitroxidative stress and subsequent vascular cell damage and vascular barrier dysfunction. The signaling cascades and sequential functional changes illustrated in Fig. 9 offer novel insight into ROS and/or RNS‐mediated pathogenesis of cardiovascular diseases.

## Conflict of Interest

The authors declare no competing financial interests.

## References

[phy214206-bib-0001] Alexander, J. S. , B. C. Alexander , L. A. Eppihimer , N. Goodyear , R. Haque , C. P. Davis , et al. 2000 Inflammatory mediators induce sequestration of VE‐cadherin in cultured human endothelial cells. Inflammation 24:99–113. 10718113 10.1023/a:1007025325451

[phy214206-bib-0002] Ames, B. N. , R. Cathcart , E. Schwiers , and P. Hochstein . 1981 Uric acid provides an antioxidant defense in humans against oxidant‐ and radical‐caused aging and cancer: a hypothesis. Proc. Natl. Acad. Sci. USA. 78:6858–6862. 6947260 10.1073/pnas.78.11.6858 PMC349151

[phy214206-bib-0003] Baluk, P. , A. Hirata , G. Thurston , T. Fujiwara , C. R. Neal , C. C. Michel , et al. 1997 Endothelial gaps: time course of formation and closure in inflamed venules of rats. Am. J. Physiol. 272:L155–170. 9038915 10.1152/ajplung.1997.272.1.L155

[phy214206-bib-0004] Banerjee, D. , U. K. Madhusoodanan , S. Nayak , and J. Jacob . 2003 Urinary hydrogen peroxide: a probable marker of oxidative stress in malignancy. Clin. Chim. Acta. 334:205–209. 12867293 10.1016/s0009-8981(03)00236-5

[phy214206-bib-0005] Cai, H. 2005 NAD(P)H oxidase‐dependent self‐propagation of hydrogen peroxide and vascular disease. Circ. Res. 96:818–822. 15860762 10.1161/01.RES.0000163631.07205.fb

[phy214206-bib-0006] Cai, H. , and D. G. Harrison . 2000 Endothelial dysfunction in cardiovascular diseases: the role of oxidant stress. Circ. Res. 87:840–844. 11073878 10.1161/01.res.87.10.840

[phy214206-bib-0007] Curry, P. E. H. V. , and I. H. Sarelius . 1983 Techniques in microcirculation: measurement of permeability, pressure and flow. Elsevier, New York.

[phy214206-bib-0008] Del Maestro, R. F. , J. Björk , and K‐E Arfors . 1981 Increase in microvascular permeability induced by enzymatically generated free radicals. II. Role of superoxide anion radical, hydrogen peroxide, and hydroxyl radical. Microvasc. Res. 22:255–270. 6276700 10.1016/0026-2862(81)90096-0

[phy214206-bib-0009] Di, A. , D. Mehta , and A. B. Malik . 2016 ROS‐activated calcium signaling mechanisms regulating endothelial barrier function. Cell. Calcium. 60:163–171. 26905827 10.1016/j.ceca.2016.02.002 PMC4988951

[phy214206-bib-0010] Dikalov, S. , K. K. Griendling , and D. G. Harrison . 2007 Measurement of reactive oxygen species in cardiovascular studies. Hypertension. 49:717–727. 17296874 10.1161/01.HYP.0000258594.87211.6b PMC1993891

[phy214206-bib-0011] Djordjevic, T. , A. Pogrebniak , R. S. BelAiba , S. Bonello , C. Wotzlaw , H. Acker , et al. 2005 The expression of the NADPH oxidase subunit p22phox is regulated by a redox‐sensitive pathway in endothelial cells. Free. Radic. Biol. Med. 38:616–630. 15683718 10.1016/j.freeradbiomed.2004.09.036

[phy214206-bib-0012] Hall, C. N. , and J. Garthwaite . 2009 What is the real physiological NO concentration in vivo? Nitric. Oxide. 21:92–103. 19602444 10.1016/j.niox.2009.07.002 PMC2779337

[phy214206-bib-0013] Hatakeyama, T. , P. J. Pappas , R. W. 2nd Hobson , M. P. Boric , W. C. Sessa , and W. N. Duran . 2006 Endothelial nitric oxide synthase regulates microvascular hyperpermeability in vivo. J. Physiol. 574. 10.1113/jphysiol.2006.108175 PMC1817804 16675496

[phy214206-bib-0014] He, P. , X. Zhang , and F. E. Curry . 1996 Ca2+ entry through conductive pathway modulates receptor‐mediated increase in microvessel permeability. Am. J. Physiol. 271:H2377–H2387. 8997296 10.1152/ajpheart.1996.271.6.H2377

[phy214206-bib-0015] He, P. , B. Liu , and F. E. Curry . 1997 Effect of nitric oxide synthase inhibitors on endothelial [Ca^2+^]i and microvessel permeability. Am. J. Physiol. 272:H176–H185. 9038936 10.1152/ajpheart.1997.272.1.H176

[phy214206-bib-0016] Hooper, D. C. , S. Spitsin , R. B. Kean , J. M. Champion , G. M. Dickson , I. Chaudhry , et al. 1998 Uric acid, a natural scavenger of peroxynitrite, in experimental allergic encephalomyelitis and multiple sclerosis. Proc. Natl. Acad. Sci. USA. 95:675–680. 9435251 10.1073/pnas.95.2.675 PMC18479

[phy214206-bib-0017] Jiang, Y. , K. Wen , X. Zhou , D. Schwegler‐Berry , V. Castranova , and P. He . 2008 Three‐dimensional localization and quantification of PAF‐induced gap formation in intact venular microvessels. Am. J. Physiol. Heart. Circ. Physiol. 295:H898–H906. 18515648 10.1152/ajpheart.00309.2008 PMC2519217

[phy214206-bib-0018] Jin, B. Y. , A. J. Lin , D. E. Golan , and T. Michel . 2012 MARCKS protein mediates hydrogen peroxide regulation of endothelial permeability. Proc. Natl. Acad. Sci. USA. 109:14864–14869. 22927426 10.1073/pnas.1204974109 PMC3443126

[phy214206-bib-0019] Jourd'heuil, D. 2002 Increased nitric oxide‐dependent nitrosylation of 4,5‐diaminofluorescein by oxidants: implications for the measurement of intracellular nitric oxide. Free. Radic. Biol. Med. 33:676–684. 12208354 10.1016/s0891-5849(02)00955-3

[phy214206-bib-0020] Kean, R. B. , S. V. Spitsin , T. Mikheeva , G. S. Scott , and D. C. Hooper . 2000 The peroxynitrite scavenger uric acid prevents inflammatory cell invasion into the central nervous system in experimental allergic encephalomyelitis through maintenance of blood‐central nervous system barrier integrity. J. Immunol. 165:6511–6518. 11086092 10.4049/jimmunol.165.11.6511

[phy214206-bib-0021] Kendall, S. , and C. C. Michel . 1995 The measurement of permeability in single rat venules using the red cell microperfusion technique. Exp. Physiol. 80:359–372. 7640005 10.1113/expphysiol.1995.sp003853

[phy214206-bib-0022] Kevil, C. G. , T. Oshima , B. Alexander , L. L. Coe , and J. S. Alexander . 2000 H_2_O_2_‐mediated permeability: role of MAPK and occludin. Am. J. Physiol. Cell. Physiol. 279:C21–C30. 10898713 10.1152/ajpcell.2000.279.1.C21

[phy214206-bib-0023] Kim, J. Y. , J. Y. Lee , K. S. Ha , E. T. Han , W. S. Park , C. K. Min , et al. 2018 Perivascular cells and NADPH oxidase inhibition partially restore hyperglycemia‐induced alterations in hematopoietic stem cell and myeloid‐derived suppressor cell populations in the bone marrow. Int. J. Stem. Cells. 12:63–72. 30595009 10.15283/ijsc18097 PMC6457702

[phy214206-bib-0024] Kuzkaya, N. , N. Weissmann , D. G. Harrison , and S. Dikalov . 2005 Interactions of peroxynitrite with uric acid in the presence of ascorbate and thiols: implications for uncoupling endothelial nitric oxide synthase. Biochem. Pharmacol. 70:343–354. 15963955 10.1016/j.bcp.2005.05.009

[phy214206-bib-0025] Lacy, F. , M. T. Kailasam , D. T. O'Connor , G. W. Schmid‐Schonbein , and R. J. Parmer . 2000 Plasma hydrogen peroxide production in human essential hypertension: role of heredity, gender, and ethnicity. Hypertension. 36:878–884. 11082160 10.1161/01.hyp.36.5.878

[phy214206-bib-0026] Lee, K. S. , S. R. Kim , S. J. Park , H. S. Park , K. H. Min , M. H. Lee , et al. 2006 Hydrogen peroxide induces vascular permeability via regulation of vascular endothelial growth factor. Am. J. Respir. Cell. Mol. Biol. 35:190–197. 16574943 10.1165/rcmb.2005-0482OC

[phy214206-bib-0027] Li, W. G. , F. J. Jr Miller , H. J. Zhang , D. R. Spitz , L. W. Oberley , and N. L. Weintraub . 2001 H_2_O_2_‐induced O_2_ production by a non‐phagocytic NAD(P)H oxidase causes oxidant injury. J. Biol. Chem. 276:29251–29256. 11358965 10.1074/jbc.M102124200 PMC3974124

[phy214206-bib-0028] Liu, S. , J. S. Beckman , and D. D. Ku . 1994 Peroxynitrite, a product of superoxide and nitric oxide, produces coronary vasorelaxation in dogs. J. Pharmacol. Exp. Ther. 268:1114–1121. 8138924

[phy214206-bib-0029] Majno G. , and Palade G. E. 1961 Studies on inflammation. 1. The effect of histamine and serotonin on vascular permeability: an electron microscopic study. J. Biophys. Biochem. Cytol. 11:571–605. 14468626 10.1083/jcb.11.3.571 PMC2225138

[phy214206-bib-0030] Mayhan, W. G. 1992 Role of nitric oxide in modulating permeability of hamster cheek pouch in response to adenosine 5'‐diphosphate and bradykinin. Inflammation. 16:295–305. 1526662 10.1007/BF00917622

[phy214206-bib-0031] Michel, C. C. , and F. E. Curry . 1999 Microvascular permeability. Physiol. Rev. 79:703–761. 10390517 10.1152/physrev.1999.79.3.703

[phy214206-bib-0032] Mundi, S. , M. Massaro , E. Scoditti , M. A. Carluccio , V. W. M. van Hinsbergh , M. L. Iruela‐Arispe , et al. 2017 Endothelial permeability, LDL deposition, and cardiovascular risk factors ‐ a review. Cardiovasc. Res. 114:35–52. 10.1093/cvr/cvx226 PMC7729208 29228169

[phy214206-bib-0033] Pacher, P. , J. S. Beckman , and L. Liaudet . 2007 Nitric oxide and peroxynitrite in health and disease. Physiol. Rev. 87:315–424. 17237348 10.1152/physrev.00029.2006 PMC2248324

[phy214206-bib-0034] Pietruczuk, P. , A. Jain , E. R. Simo‐Cheyou , M. B. Anand‐Srivastava , and A. K. Srivastava . 2019 Protein kinase B/AKT mediates insulin‐like growth factor 1‐induced phosphorylation and nuclear export of histone deacetylase 5 via NADPH oxidase 4 activation in vascular smooth muscle cells. J. Cell. Physiol. 234:17337–17350. 30793765 10.1002/jcp.28353

[phy214206-bib-0035] Prutz, W. A. , H. Monig , J. Butler , and E. J. Land . 1985 Reactions of nitrogen dioxide in aqueous model systems: oxidation of tyrosine units in peptides and proteins. Arch. Biochem. Biophys. 243:125–134. 4062299 10.1016/0003-9861(85)90780-5

[phy214206-bib-0036] Radi, R. 2004 Nitric oxide, oxidants, and protein tyrosine nitration. Proc. Natl. Acad. Sci. USA. 101:4003–4008. 15020765 10.1073/pnas.0307446101 PMC384685

[phy214206-bib-0037] Radi, R. 2013 Protein tyrosine nitration: biochemical mechanisms and structural basis of functional effects. Acc. Chem. Res. 46:550–559. 23157446 10.1021/ar300234c PMC3577981

[phy214206-bib-0038] Radi, R. , J. S. Beckman , K. M. Bush , and B. A. Freeman . 1991 Peroxynitrite oxidation of sulfhydryls. The cytotoxic potential of superoxide and nitric oxide. J. Biol. Chem. 266:4244–4250. 1847917

[phy214206-bib-0039] Reiter, C. D. , R. J. Teng , and J. S. Beckman . 2000 Superoxide reacts with nitric oxide to nitrate tyrosine at physiological pH via peroxynitrite. J. Biol. Chem. 275:32460–32466. 10906340 10.1074/jbc.M910433199

[phy214206-bib-0040] Riobo, N. A. , E. Clementi , M. Melani , A. Boveris , E. Cadenas , S. Moncada , et al. 2001 Nitric oxide inhibits mitochondrial NADH:ubiquinone reductase activity through peroxynitrite formation. Biochem. J. 359:139. 11563977 10.1042/0264-6021:3590139 PMC1222129

[phy214206-bib-0041] Rutledge, J. C. , F. R. Curry , J. F. Lenz , and P. A. Davis . 1990 Low density lipoprotein transport across a microvascular endothelial barrier after permeability is increased. Circ. Res. 66:486–495. 1688742 10.1161/01.res.66.2.486

[phy214206-bib-0042] Rutledge, J. C. , F. E. Curry , P. Blanche , and R. M. Krauss . 1995 Solvent drag of LDL across mammalian endothelial barriers with increased permeability. Am. J. Physiol. 268:H1982–1991. 7771548 10.1152/ajpheart.1995.268.5.H1982

[phy214206-bib-0043] Sanchez, F. A. , N. B. Savalia , R. G. Duran , B. K. Lal , M. P. Boric , and W. N. Duran . 2006 Functional significance of differential eNOS translocation. Am. J. Physiol. Heart. Circ. Physiol. 291:H1058–H1064. 16679407 10.1152/ajpheart.00370.2006 PMC1629085

[phy214206-bib-0044] Shen, Y. H. , X. L. Wang , and D. E. Wilcken . 1998 Nitric oxide induces and inhibits apoptosis through different pathways. FEBS. Lett. 433:125–131. 9738946 10.1016/s0014-5793(98)00844-8

[phy214206-bib-0045] Shimizu, S. , M. Nomoto , S. Naito , T. Yamamoto , and K. Momose . 1998 Stimulation of nitric oxide synthase during oxidative endothelial cell injury. Biochem. Pharmacol. 55:77–83. 9413933 10.1016/s0006-2952(97)00399-7

[phy214206-bib-0046] Suresh, K. , L. Servinsky , J. Reyes , S. Baksh , C. Undem , M. Caterina , et al. 2015 Hydrogen peroxide‐induced calcium influx in lung microvascular endothelial cells involves TRPV4. Am. J. Physiol. Lung. Cell. Mol. Physiol. 309:L1467–1477. 26453519 10.1152/ajplung.00275.2015 PMC4683318

[phy214206-bib-0047] Szabo, C. , H. Ischiropoulos , and R. Radi . 2007 Peroxynitrite: biochemistry, pathophysiology and development of therapeutics. Nat. Rev. Drug. Discov. 6:662–680. 17667957 10.1038/nrd2222

[phy214206-bib-0048] Tsukada, K. , T. Hasegawa , S. Tsutsumi , H. Katoh , H. Kuwano , T. Miyazaki , et al. 2000 Effect of uric acid on liver injury during hemorrhagic shock. Surgery. 127:439–446. 10776436 10.1067/msy.2000.104486

[phy214206-bib-0049] Villa, L. M. , E. Salas , V. M. Darley‐Usmar , M. W. Radomski , and S. Moncada . 1994 Peroxynitrite induces both vasodilatation and impaired vascular relaxation in the isolated perfused rat heart. Proc. Natl. Acad. Sci. USA. 91:12383–12387. 7809045 10.1073/pnas.91.26.12383 PMC45442

[phy214206-bib-0050] Walford, G. A. , R. L. Moussignac , A. W. Scribner , J. Loscalzo , and J. A. Leopold . 2004 Hypoxia potentiates nitric oxide‐mediated apoptosis in endothelial cells via peroxynitrite‐induced activation of mitochondria‐dependent and ‐independent pathways. J. Biol. Chem. 279:4425–4432. 14597620 10.1074/jbc.M310582200

[phy214206-bib-0051] Wardman, P. 2007 Fluorescent and luminescent probes for measurement of oxidative and nitrosative species in cells and tissues: progress, pitfalls, and prospects. Free. Radic. Biol. Med. 43:995–1022. 17761297 10.1016/j.freeradbiomed.2007.06.026

[phy214206-bib-0052] Wu, N. Z. , and A. L. Baldwin . 1992 Transient venular permeability increase and endothelial gap formation induced by histamine. Am. J. Physiol. 262:H1238–H1247. 1566906 10.1152/ajpheart.1992.262.4.H1238

[phy214206-bib-0053] Xia, Y. , V. L. Dawson , T. M. Dawson , S. H. Snyder , and J. L. Zweier . 1996 Nitric oxide synthase generates superoxide and nitric oxide in arginine‐depleted cells leading to peroxynitrite‐mediated cellular injury. Proc. Natl. Acad. Sci. USA. 93:6770–6774. 8692893 10.1073/pnas.93.13.6770 PMC39102

[phy214206-bib-0054] Yuan, Y. , H. J. Granger , D. C. Zawieja , D. V. DeFily , and W. M. Chilian . 1993 Histamine increases venular permeability via a phospholipase C‐NO synthase‐guanylate cyclase cascade. Am. J. Physiol. 264:H1734–H1739. 7684577 10.1152/ajpheart.1993.264.5.H1734

[phy214206-bib-0055] Zhou, X. , and P. He . 2010 Endothelial [Ca2+]i and caveolin‐1 antagonistically regulate eNOS activity and microvessel permeability in rat venules. Cardiovasc. Res. 87:340–347. 20080986 10.1093/cvr/cvq006 PMC2895537

[phy214206-bib-0056] Zhou, X. , and P. He . 2011 Improved measurements of intracellular nitric oxide in intact microvessels using 4,5‐diaminofluorescein diacetate. Am. J. Physiol. Heart. Circ. Physiol. 301:H108–H114. 21536843 10.1152/ajpheart.00195.2011 PMC3129913

[phy214206-bib-0057] Zhou, X. , and P. He . 2011 Temporal and spatial correlation of platelet activating factor‐induced increases in endothelial [Ca2+]i, nitric oxide, and gap formation in intact venules. Am. J. Physiol. Heart. Circ. Physiol. 301:H108–14. 21873500 10.1152/ajpheart.00599.2011 PMC3213973

[phy214206-bib-0058] Zhou, X. , K. Wen , D. Yuan , L. Ai , and P. He . 2009 Calcium influx‐dependent differential actions of superoxide and hydrogen peroxide on microvessel permeability. Am. J. Physiol. Heart. Circ. Physiol. 296:H1096–H1107. 19201997 10.1152/ajpheart.01037.2008 PMC2670695

[phy214206-bib-0059] Zhou, X. , D. Yuan , M. Wang , and P. He . 2013 H2O2‐induced endothelial NO production contributes to vascular cell apoptosis and increased permeability in rat venules. Am. J. Physiol. Heart. Circ. Physiol. 304:H82–H93. 23086988 10.1152/ajpheart.00300.2012 PMC3543683

[phy214206-bib-0060] Zhu, L. , and P. He . 2005 Platelet‐activating factor increases endothelial [Ca^2+^]i and NO production in individually perfused intact microvessels. Am. J. Physiol. Heart. Circ. Physiol. 288. 10.1152/ajpheart.01080.2004 15665052

[phy214206-bib-0061] Zhu, L. , and P. He . 2006 fMLP‐stimulated release of reactive oxygen species from adherent leukocytes increases microvessel permeability. Am. J. Physiol. Heart. Circ. Physiol. 290:H365–H372. 16155097 10.1152/ajpheart.00812.2005

[phy214206-bib-0062] Zielonka, J. , J. Vasquez‐Vivar , and B. Kalyanaraman . 2008 Detection of 2‐hydroxyethidium in cellular systems: a unique marker product of superoxide and hydroethidine. Nat. Protoc. 3:8–21. 18193017 10.1038/nprot.2007.473

[phy214206-bib-0063] Zou, M. H. , X. Y. Hou , C. M. Shi , D. Nagata , K. Walsh , and R. A. Cohen . 2002 Modulation by peroxynitrite of Akt‐ and AMP‐activated kinase‐dependent Ser1179 phosphorylation of endothelial nitric oxide synthase. J. Biol. Chem. 277:32552–32557. 12107173 10.1074/jbc.M204512200

[phy214206-bib-0064] Zou, M. H. , C. Shi , and R. A. Cohen . 2002 Oxidation of the zinc‐thiolate complex and uncoupling of endothelial nitric oxide synthase by peroxynitrite. J. Clin. Invest. 109:817–826. 11901190 10.1172/JCI14442 PMC150913

